# QTLMAS 2010: simulated dataset

**DOI:** 10.1186/1753-6561-5-S3-S3

**Published:** 2011-05-27

**Authors:** Maciej Szydlowski, Paulina Paczyńska

**Affiliations:** 1 Department of Genetics and Animal Breeding, Poznan University of Life Sciences, Wolynska 33, 60-637 Poznan, Poland

## Abstract

**Background:**

Objective was to simulate the data for the QTLMAS 2010 Workshop under a model that includes major additive, epistatic and parent-of-origin effects.

**Results:**

Data were simulated for 3226 individuals in 5 generations. Genomic data for 5 chromosomes were simulated using coalescent model. In total, the data included 10,031 SNPs, 30 additive QTLs, 2 interacting QTL pairs, and 3 imprinted loci. The density was 20 SNPs/1Mb, whereas mean linkage disequilibrium between adjacent SNPs was 0.1. One quantitative and one binary trait were simulated with heritability of 0.39-0.52 and additive correlation of 0.59. The data can be used as a benchmark for comparison of QTL mapping methods and models for genomic breeding value estimation under complex genetic architecture.

## Background

Methods for genome marker assisted selection are being developed for many livestock species. Models focus mainly on additive genes, whereas other effects like epistasis or epigenetic effects are usually ignored. However, epistatic interactions have been documented in model and livestock species, and can contribute significantly to phenotype variation [1]. Studies in mammals highlights the importance of imprinting in regulating development and metabolism. Accounting for significant non-additive and non-mendelian effects may increase power of the method for QTL mapping and the accuracy of estimated breeding values. Furthermore, widespread pleiotropy is observed when large numbers of genes affect each trait. This suggests that multitrait models should perform better than testing one trait at time. Objective was to simulate phenotypes for two correlated traits under a model that includes epistatic and parent-of-origin effects for QTLMAS 2010 Workshop.

## Simulation method

### Pedigree

The simulated pedigree consisted of 3226 individuals in 5 generations. There were 20 founders: 5 males and 15 females. The pedigree structure was created assuming that each female mates once and gives birth to approximately 30 progeny. The parents for every next generation were selected randomly, mainly from the current generation and with some small probability from older generation, in consequence almost non-overlapping generations were created.

### Simulated genomes

Five autosomal chromosomes were simulated, each about 100M bp long. The biallelic SNP data was simulated using the ms software [2]. First, a pool of 500 haplotypes was simulated assuming an effective population size of 5000, a mutation rate of 10^-8^ per base per generation and a recombination rate of 1cM/Mb. Then we randomly selected polymorphic SNPs (MAF>0.1) from the pool to achieve density of approx. 20 SNPs/Mb with minimum distance 50 bp. The genomes for founders were compiled by drawing a pair of haplotypes from the haplotype pool. Due to the limited number of founders, some polymorphisms observed in the haplotype pool was lost. The founders' alleles were then dropped down the pedigree with assumed recombination rate. No recombination hotspot were created.

### Simulated phenotypes

We simulated two complex phenotypic traits: a quantitative trait (QT) and a binary trait (BT), determined by 37 QTLs, including 9 controlled genes and 28 random genes. All QTLs were selected from previously simulated SNPs and the genotypes for 28 QTLs were then removed from the data. The controlled genes for QT were selected based on their high polymorphism (MAF 0.44-0.50) and high LD with markers. The controlled QTLs included two pairs of epistatic genes with no individual effects, 3 maternally imprinted genes and two additive major genes. The random genes for QT were drawn from the simulated SNPs excluding chromosome 5, whereas their additive effects were sampled from the normal distribution. A random subset of the additive QTLs was assumed to have pleiotropic effect on BT. Residuals were assumed uncorrelated and sampled from normal distribution with variance 51.76 and 18.16 for QT and BT, respectively. The threshold for BT was set to 9.22.

## Simulation results

The simulation algorithm produced 10'031 markers (chromosomes 1-5: 1976, 2058, 2048, 1991, 1958), including 263 monomorphic (polymorphism lost due to bottleneck) and 9'768 biallelic SNPs. We calculated allele frequency by simply counting genotypes for 3226 individuals. The minor allele frequency (MAF) for polymorphic SNPs ranged from 0.002 to 0.5 with the mean value of 0.299 (MAF<0.1 - 948 SNPs, 0.1≤MAF<0.2 - 1601, 0.2≤MAF<0.3 - 1948, 0.3≤MAF<0.4 - 2479, MAF≥0.4 - 2792). Out of the 9345 SNPs with MAF>0.05, 3933 loci showed significant deviation from Hardy-Weinberg (HW) equilibrium (Pearson test under individual test error rate of 1%). Concerning the 3933 SNPs half the deviation from HW equilibrium for heterozygous genotype was 47 individuals on average.

**Figure 1:**
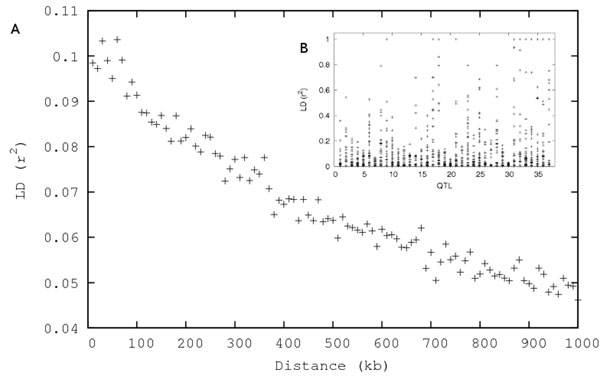
**Linkage disequilibrium of simulated data. A.** Mean LD (r^2^) between marker SNPs versus physical distance. **B.** LD between each QTL and markers located within 1Mb distance from the QTL.

The distance between adjacent SNPs ranged from 59 to 625'374 bp with the mean 49'761.7 bp (SD=55'191.2 bp). Considerable amount of linkage disequilibrium (LD) was created (Fig. 1). Mean LD (r^2^ calculated from unphased genotypes) between adjacent SNPs with MAF>0.05 was 0.100 (SD=0.152), however, the average LD between close SNPs was relatively low when compared to human, dog or cattle. Each simulated QTL was surrounded by 19-47 polymorphic SNPs (MAF>0.05) located within 1Mb distance from the QTL.

Epistatic QTLs contributed 11% of phenotypic variance for QT, whereas imprinted genes explained 6.5%. In case of QT, the true breeding value (TBV) was calculated as the sum of 30 additive QTLs, haplotype effects of epistatic QTLs and the effects of imprinted QTLs (for males only), whereas TBV for BT was the sum of 22 additive QTLs. The narrow-sense heritability (h^2^) for QT was 0.52 for males and 0.39 for females, whereas h^2^ for BT was 0.48. The correlation between breeding values for the two traits was 0.59 for males and 0.68 for females.

## Discussion

Our simulated data were intended for comparison of new approaches to QTL mapping and marker based genetic evaluation. These applications depend on LD between QTLs and markers, which was created here by coalescent population genetic model. It was shown that this model can faster produce data that closely resemble empirical data in LD pattern [3]. Single sample of genome is insufficient to study all properties of potential methods (e.g. power of QTL identification), however, due to huge amount of computations usually involved in an analysis of entire genome, multiple samples are rarely feasible.

The density of simulated markers (approx. 20 SNPs/Mb) was close to current genotyping platform for bovines (17 SNPs/Mb) and lower than chickens (35 SNPs/Mb), however the total number of markers was low. This small genome size can be treated as a subsample from a real whole-genome study in mammal.

The epistatic and imprinted controlled genes added to overall genetic complexity of QT, whereas the pleiotropic genes created correlation between the two phenotypes. Epistatic QTLs are mainly studied in model organisms and within designed crosses. Here, the frequency of the simulated epistatic QTLs and limited LD were optimal for the even distribution of two-locus genotypes. Interacting loci were limited to paired SNPs because higher-order epistasis, which require much larger samples is impractical to test. Because the QTLs have no individual effects, they are difficult to catch in multistep procedure, in which important SNPs are first selected based on single locus properties. The simulated epistatic effects were of additive by additive type, which, after the dominance-by-dominance interaction, was the most prevalent in recent study on pigs [1].

There is accumulating evidence that polymorphisms within imprinted genes are associated with muscle mass, fat deposition, growth and milk production in livestock [4]. Their individual effects were detectable within the genotyped population (N=2326) if individual SNPs were tested under true (maternal imprinting) model from phased genotypes (P<1.98×10^–7^). The density of SNPs and the amount of LD will allow to easily recover haplotypes in the available data.

Genetic correlation between the two traits was sufficient to study potential advantage from using multitrait models. To create clear pattern of the correlation, pleiotropic effects of different genes were all in the same direction. The limited LD between adjacent loci should allow to disentangle pleitropy from close linkage.

## Remarks

QTL positions and their effects as well as the censored and complete data are available at http://jay.up.poznan.pl/qtlmas2010.

## Authors' contribution

MS programmed the simulation. MS and PP wrote the manuscript.

## Competing interests

Authors declare no competing interests.
